# Genome-wide identification of *BBX* gene family and their expression patterns under salt stress in soybean

**DOI:** 10.1186/s12864-022-09068-5

**Published:** 2022-12-12

**Authors:** Binghui Shan, Guohua Bao, Tianran Shi, Lulu Zhai, Shaomin Bian, Xuyan Li

**Affiliations:** College of Plant Science, Jilin University, Changchun, China

**Keywords:** Soybean, BBX family, Phylogenetic evolution, Salt stress, Gene expression, Prediction of protein interaction, Binding sites of transcription factor

## Abstract

**Background:**

*BBX* genes are key players in the regulation of various developmental processes and stress responses, which have been identified and functionally characterized in many plant species. However, our understanding of BBX family was greatly limited in soybean.

**Results:**

In this study, 59 *BBX* genes were identified and characterized in soybean, which can be phylogenetically classified into 5 groups. *GmBBX*s showed diverse gene structures and motif compositions among the groups and similar within each group. Noticeably, synteny analysis suggested that segmental duplication contributed to the expansion of GmBBX family. Moreover, our RNA-Seq data indicated that 59 *GmBBX*s showed different transcript profiling under salt stress, and qRT-PCR analysis confirmed their expression patterns. Among them, 22 *GmBBX*s were transcriptionally altered with more than two-fold changes by salt stress, supporting that *GmBBX*s play important roles in soybean tolerance to salt stress. Additionally, Computational assay suggested that GmBBXs might potentially interact with GmGI3, GmTOE1b, GmCOP1, GmCHI and GmCRY, while eight types of transcription factors showed potentials to bind the promoter regions of *GmBBX* genes.

**Conclusions:**

Fifty-nine *BBX* genes were identified and characterized in soybean, and their expression patterns under salt stress and computational assays suggested their functional roles in response to salt stress. These findings will contribute to future research in regard to functions and regulatory mechanisms of soybean *BBX* genes in response to salt stress.

**Supplementary Information:**

The online version contains supplementary material available at 10.1186/s12864-022-09068-5.

## Introduction

Zinc finger transcription factors (TFs) constitute one of the most important and largest gene families in plants (approximately 15% of the total), which can be divided into multiple subfamilies based on their structures and functions [1]. B-box (BBX) genes constitute a subfamily of zinc-finger TF family, and they exist in all eukaryotic genomes [2, 3]. BBX proteins usually harbor one or two B-box domain(s) required for transcriptional regulation and protein-protein interaction in the N-terminal region [2–4]. According to the consensus sequence and the spacing feature of zinc-binding residues, B-box domains can be grouped into two types, B-box1 (C-X2-C-X7-8-C-X2-D-X-A-X-L-C-X2-C-D-X3-HB) and B-box2 (C-X2-C-X3-P-X4-C-X2-D-X3-L-C-X2-C-D-X3-H) [2–4]. Besides the B-box domains, a number of BBX proteins also contain a CCT domain (CONSTANS, CO-like and TOC1) at the C-terminal, which is involved in transcriptional regulation and nuclear transport [2, 3, 5].

The first plant *BBX* gene, *CONSTANS* (*CO*), was identified in Arabidopsis, which is involved in the regulation of photoperiodic flowering [6]. With the availability of complete plant genomic sequences, a considerable number of *BBX* genes have been isolated in many plant species. For example, 32 *BBX* genes were identified in Arabidopsis [3], 29 in tomato [7], 30 in rice [5], 27 in Moso bamboo [8], 64 in apple [9], 25 in pear [10], 51 in strawberry [11], and 24 in grapevine [12], etc. Among them, Arabidopsis BBX family has been best-studied in physiological and molecular functions, which can be divided into five groups according to their domain structures [2, 3]: Group I (AtBBX1–6) and II (AtBBX7–13) harboring two B-box domains and one CCT domain, Group III (AtBBX14–17) possessing a single B-box domain and a CCT domain, Group IV (AtBBX18–25) containing two B-box domains without CCT domain; while Group V (AtBBX26–32) only showing a single B-box domain. Increasing studies indicated that different members of *BBX* family, even in the same group, perform diverse or converse functions. For example, *AtBBX2* (*AtCOL1*) and *AtBBX3* (*AtCOL2*) showed less effect on flowering [13], but altered two specific circadian rhythms; *AtBBX6* (*AtCOL5*) and *AtBBX7* (*AtCOL9*) acted as short day condition (SD)-specific inducer of flowering and long day condition (LD)-specific inhibitor of flowering, respectively [14, 15]. Similarly, *AtBBX18* (*DBB1a*), *AtBBX19* (*DBB1b*), *AtBBX24* (*STO*) and *AtBBX25* (*STH1*) acted as negative regulators to respond to light signal [16, 17], but *AtBBX21* (*STH2*) and *AtBBX22* (*LZF1/STH3*) as positive players responsive for light signal [18, 19]. Moreover, several evidence showed that orthologs of *BBX* genes in different species might play distinct roles. For instance, *AtCO* (also known as *AtBBX1*) promoted flowering under LD condition but not under SD condition [20], whereas *OsHd1* (*HEADING DATE 1*)/*OsBBX18*, the rice *CO* ortholog, contributed to rice flowering under inductive SD condition [21]. Thus, addressing the diversity of BBX family in different crop species is an important step to precisely utilize them for the improvement of agronomic traits.

*BBX* genes are crucial players in regulatory networks underlying biological and developmental processes as well as stress responses [2–4]. Noticeably, increasing evidence indicated that *BBX*s may play important roles in plant tolerance to salt stress. It was observed that the expressions of *BBX* genes were altered by salt stress. For example, five *BBX* genes in rice (*OsBBX1*, *OsBBX2*, *OsBBX8*, *OsBBX19* and *OsBBX24*) were transcriptionally induced by salt, drought and cold stresses [22], while the expression patterns of 25 *BBX* genes were changed in the roots and shoots of rapeseed [23]. More convincingly, genetic evidence showed that *BBX* genes were involved in plant response to salt stress. For instance, overexpression of *AtSTO* (*AtBBX24*) promoted root growth at high salinity in Arabidopsis [24]. Overexpression of Ginkgo *BBX25* in Populus improved salt tolerance [25], while *CpBBX19* (*Chimonanthus praecox*) conferred salt tolerance in Arabidopsis [26]. Similarly, overexpression of *IbBBX24* enhanced salt tolerance of sweet potato [27], while *MdBBX1* transgenic plants showed higher survival rate under salt stress relative to control [28]. Thus, comprehensive characterization of salt-responsive *BBX* genes is of great significance to improve salt tolerance of crop plants.

Soybean (*Glycine max*) is acknowledged as an important agricultural crop in the world owing to the rich sources of protein and edible oil. Soybean is susceptible to salt, and soil salinity can hamper plant growth and reduce crop productivity [29]. Previous study showed that 28 *CO-like BBX* genes were identified in the soybean genome, and several *BBX* genes were involved in the regulation of flowering and light-controlled development [30, 31]. However, the functional roles of soybean BBX family remain unknown in response to salt stress. In this study, 59 *BBX* genes were identified and characterized in soybean. Subsequently, their molecular evolution, gene structures and motif compositions were investigated. Furthermore, transcriptomic analysis was performed to examine the expression patterns of *BBX* genes under salt stress. Additionally, interactors of the salt-responsive BBX proteins and the binding of transcription factor were computationally surveyed. The results suggested that *GmBBX*s play important roles in soybean tolerance to salt stress, which provided a framework for understanding soybean BBX family and their response to salt stress.

## Results

### Identification of BBX family in soybean and their evolutionary relationship

To identify the *BBX* genes in soybean (*GmBBX*s), we conducted a Hidden Markov Model (HMM) search using the B-box zinc finger domain (Pfam; PF00643) against the soybean protein database (*Glycine max* Wm82.a2.v1) in Phytozome. Also, the protein sequences of Arabidopsis BBX family were used to identify GmBBXs against the above soybean protein database. Subsequently, B-box domain was further investigated using the online tools, SMART and CDD. Consequently, 59 putative *BBX* genes were identified in soybean and designated as *GmBBX*s according to the nomenclature of their corresponding *BBX* genes in Arabidopsis. The detailed information of the 59 *GmBBX*s is listed in Table S1. Briefly, the deduced proteins possessed 99 to 480 amino acids, and their molecular weights varied from 11.0 to 53.9 kDa. The isoelectric points of the deduced proteins ranged from 4.20 to 9.84. The subcellular localization was predicted using the online tool, WoLF PSORT (https://www.genscript.com/wolf-psort.html?src=leftbar), and it showed that 40 GmBBX proteins were located in nucleus, 15 in chloroplast and 4 in cytoplasm, suggesting that these *GmBBX* genes might have diverse functional roles and distinct expressions in different tissues.

**Fig. 1:**
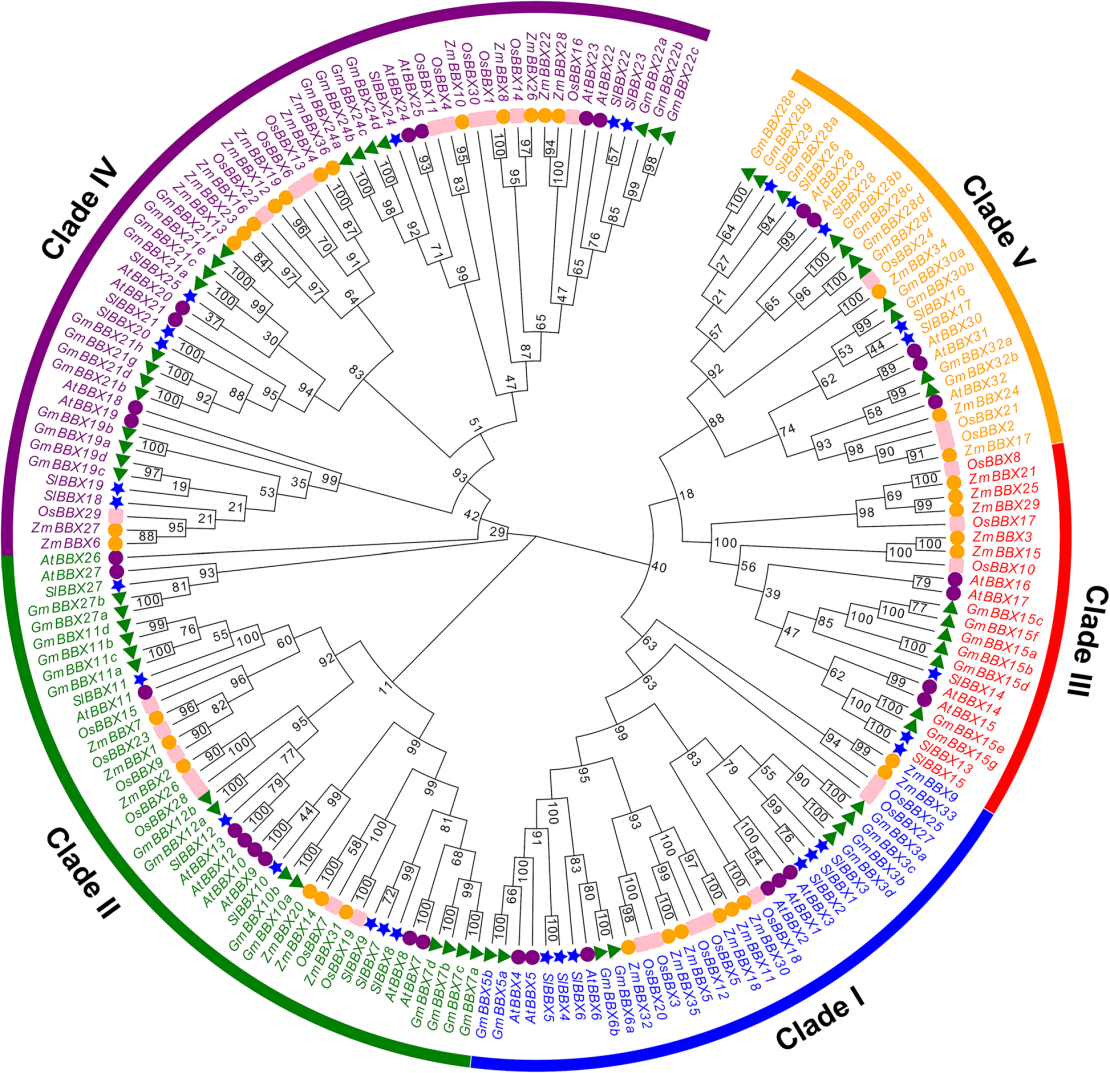
Phylogenetic analysis of BBX proteins from soybean, Arabidopsis, tomato, maize and rice. The phylogenetic tree was calculated based on protein matrix using MEGA11 and divided into five clades (I-V) with different colors. The bootstrap values are showed at each node. Green triangles, blue stars, purple dots, orange dots and pink rectangles indicate the BBX proteins of soybean (GmBBXs), tomato (SlBBXs), Arabidopsis (AtBBXs), maize (ZmBBXs) and rice (OsBBXs), respectively. The protein sequences of BBXs in the five plant species were downloaded from the Phytozome or TAIR

To analyze the evolutionary relationship of GmBBXs, a total of 186 BBX proteins, including 59 soybean BBXs, 29 tomato BBXs, 32 Arabidopsis BBXs, 36 maize BBXs, and 30 rice BBXs, were used to construct phylogenetic tree. As shown in Fig. 1, all the BBXs were divided into 5 clades, consistent with the previous studies in Arabidopsis and rice [3, 5]. GmBBXs were unevenly distributed in the five different clades. For example, 19 GmBBXs were present at the clade IV with AtBBX18–25, 8 SlBBXs and 10 OsBBXs, which might be involved in response to light signal, carotenoid biosynthesis, and stress [2, 3, 5, 32]. Eight GmBBXs were clustered together with AtBBX1–6 and six OsBBXs, which were reported to regulate flowering and/or circadian clock [2, 3, 5].

### Chromosomal distribution and expansion of BBX family in soybean

**Fig. 2:**
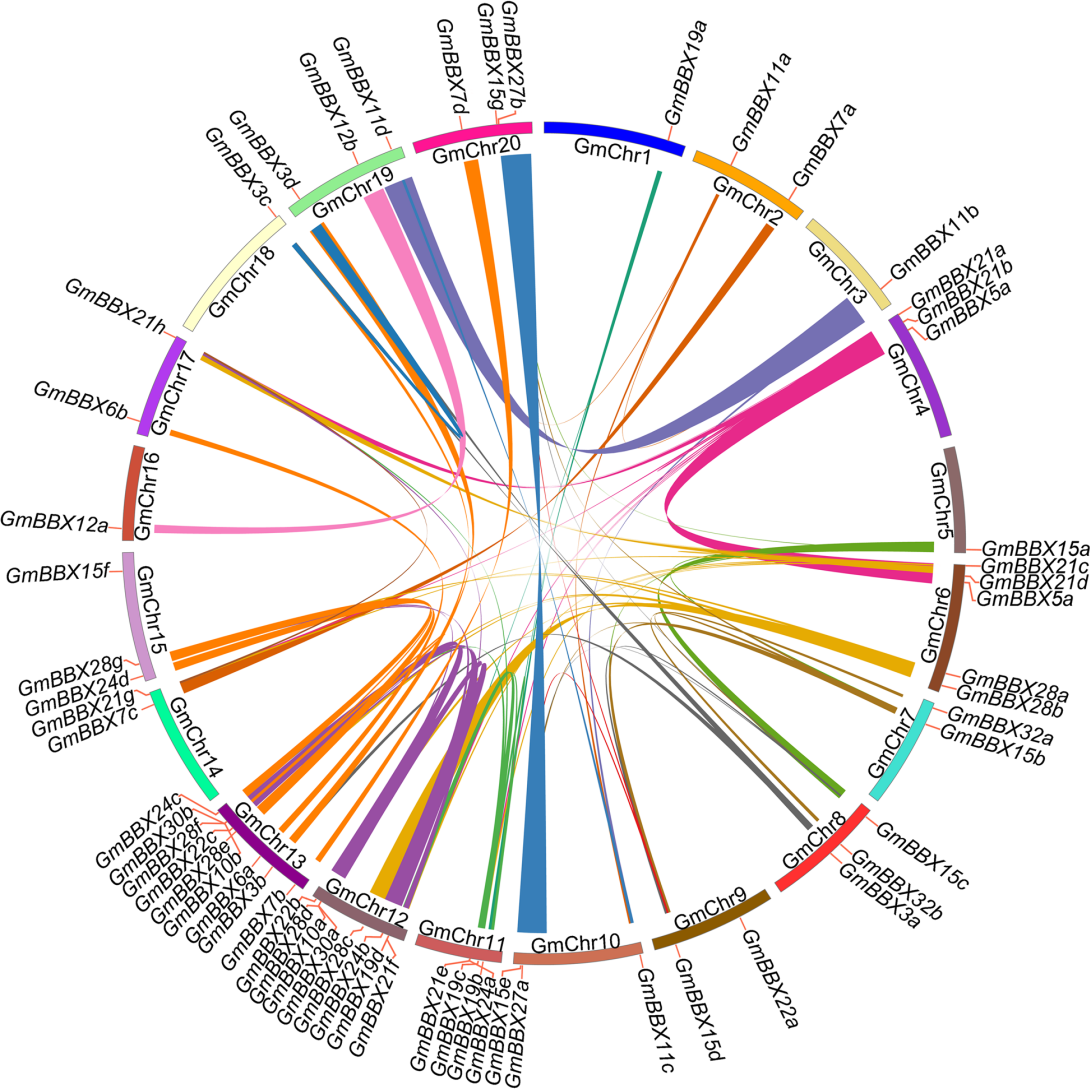
Distribution and synteny analysis of *GmBBX* genes on soybean chromosomes. The positions of the *BBX* genes on the chromosomes are shown on the outside. The colored boxes indicate the different chromosomes (GmChr1-GmChr20). Thirty-eight duplication sets covering 56 *GmBBX* genes were mapped on distinct duplicate blocks, and the colored lines connecting genes from different chromosomes represent segmental duplication events related to *GmBBX* genes

Based on the annotated genomic locations, 59 *BBX* genes were widely distributed in 20 chromosomes (Fig. 2). The chromosome 13 had the maximum amount of *GmBBX* genes (nine), followed by the chromosome 12 with eight *BBX* genes and the chromosome 6 with five *BBX* genes. Seven chromosomes (the chromosomes 4, 8, 10, 11, 15, 19 and 20) harbored three *BBX* genes. The chromosomes 2, 7, 9, 14 and 17 contained two *GmBBX* genes, only one *GmBBX* gene was observed in the chromosomes 1, 3, 5, 16 and 18.

**Fig. 3:**
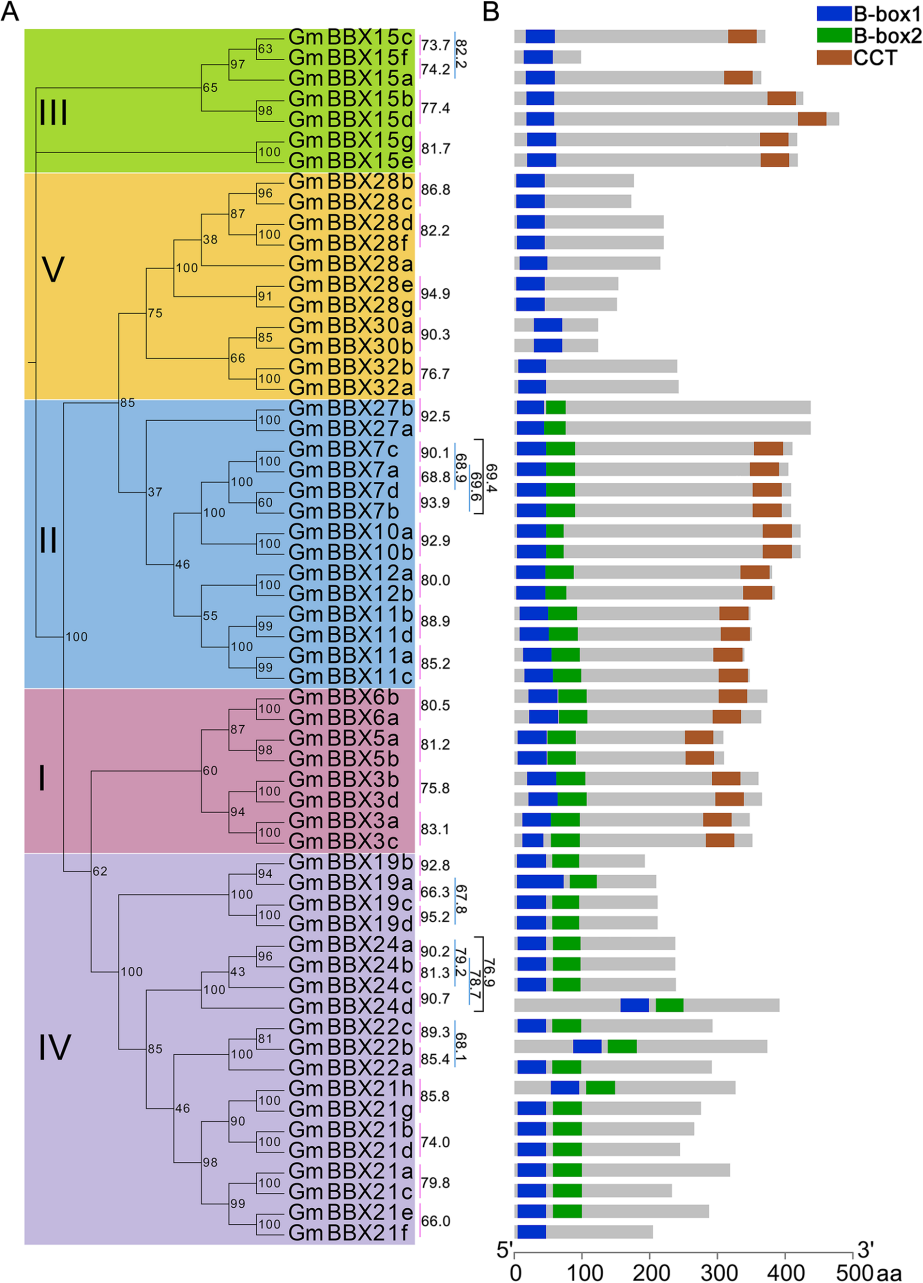
Phylogenetic analysis and conserved structural domains of GmBBX proteins. **A** Phylogenetic analysis of BBX proteins in soybean using MEGA11. The Roman numerals (I-V) indicate the five groups, and the numbers to the right of the phylogenetic tree indicate the percentage identity between two GmBBX proteins. **B** The diagrams of conserved domains for the 59 GmBBX proteins. The length of each protein sequence is represented by the grey bar. The colored boxes refer to the conserved domains: brown box, CCT domain; dark blue box, B-box1 domain; green box, B-box2 domain. The sequence length of each protein is represented by grey bar at the bottom

It is well accepted that segmental and tandem duplication are two important ways to expand gene family [33]. Thus, we surveyed if segmental duplication contributed to the formation of soybean BBX family. As shown in Fig. 2 and Table S2, 38 duplication sets covering 56 *GmBBX* genes were mapped on distinct duplicate blocks. Noticeably, the 38 duplication sets were clustered into a discrete clade in phylogenetic tree with high protein identity for example GmBBX7a/7c (90.10%), GmBBX7b/7d (93.89%), GmBBX10a/10b (92.86%), GmBBX19a/19b (92.82%), GmBBX19c/19d (95.24%), GmBBX24a/24b (90.21%), GmBBX24c/24d (90.72%), GmBBX27a/27b (92.47%), GmBBX28e/28 g (94.93%), GmBBX30a/30b (90.32%) (Fig. 3A and Table S3). Tandem duplication generally refers to two paralogs separated by five genes or less on the same chromosome. However, no tandem duplication was observed for GmBBX family. These observations suggested that BBX family possibly arose from segmental duplication rather than tandem duplication in soybean.

Furthermore, syntenic relationship of *BBX* genes was estimated between Arabidopsis and soybean. Consequently, 54 orthologous *BBX* gene pairs were observed between the two species, comprising 41 *GmBBX*s and 17 *AtBBX*s (Fig. S1 and Table S4). These observations suggested that most of the Gm*BBX* genes had appeared before the evolutional divergence of soybean and Arabidopsis. Noticeably, genomic synteny analysis was shown between *GmBBX24d* and a non-*BBX* gene *AT2G43390*, the C-terminal of which is very similar to the N-terminal of *GmBBX24d*.

To investigate potential selective pressure for *GmBBX* gene duplication events, we calculated the nonsynonymous (Ka) and synonymous (Ks) substitution ratios (Ka/Ks). The Ka/Ks values of the duplicated gene pairs were less than 1 (0.048–0.469) between soybean BBX genes (Table S2), and 0.068–0.209 between soybean and Arabidopsis (Table S4), suggesting that they evolved under the purifying selection. Furthermore, it was estimated that the duplication events between soybean *BBX* genes might occur at 6.34 to 299.23 million years ago (MYA) (Table S2), and 121.54 to 401.58 MYA between soybean and Arabidopsis (Table S4).

### Diverse motif compositions of GmBBX family and their gene structures

Fifty-nine GmBBX proteins were divided into 5 clades in phylogenetic tree (Fig. 3A). BBX proteins in the clade I (eight members) and the clade II (14 members) contained two B-box domains and one CCT domain (Fig. 3). Additionally, the proteins in the clade II harbored a relatively conserved amino acid sequence (SANPLASR) and a VP-motif (Fig. S2). The clade III comprised seven BBX proteins with one B-box domain and one CCT domain (Fig. 3). The proteins in the clade IV (19 members) and clade V (11 members) only possessed two B-box domains and one B-box domain, respectively (Fig. 3). Unlike other BBXs in the clade II, however, GmBBX27a and GmBBX27b only contained two B-box domains without CCT domain (Fig. 3B). Meanwhile, GmBBX15f was lack of CCT domain in the clade III, and GmBBX21f only possessed B-box1 without B-box2 in the clade IV (Fig. 3B). It was observed that the B-box1 and B-box2 domains of the 59 GmBBX proteins were C-X2-C-X8-C-X2-D(H)-X-A-X-L-C-X2-C-D-X3-H-X2-N-X5-H and C-X2-C-X4-A(G)-X3-C-X7-C-D-X3-H(N)-X8-H, respectively (Fig. S3). In addition, the CCT domains of 26 soybean BBX proteins showed a highly conserved sequence R-X5-R-Y-X2-K-X3-R-X3-K-X2-R-Y-X2-R-K-X2-A-X2-R-X-R-X2-G-R-F-X-K(R) (Fig. S3).

**Fig. 4:**
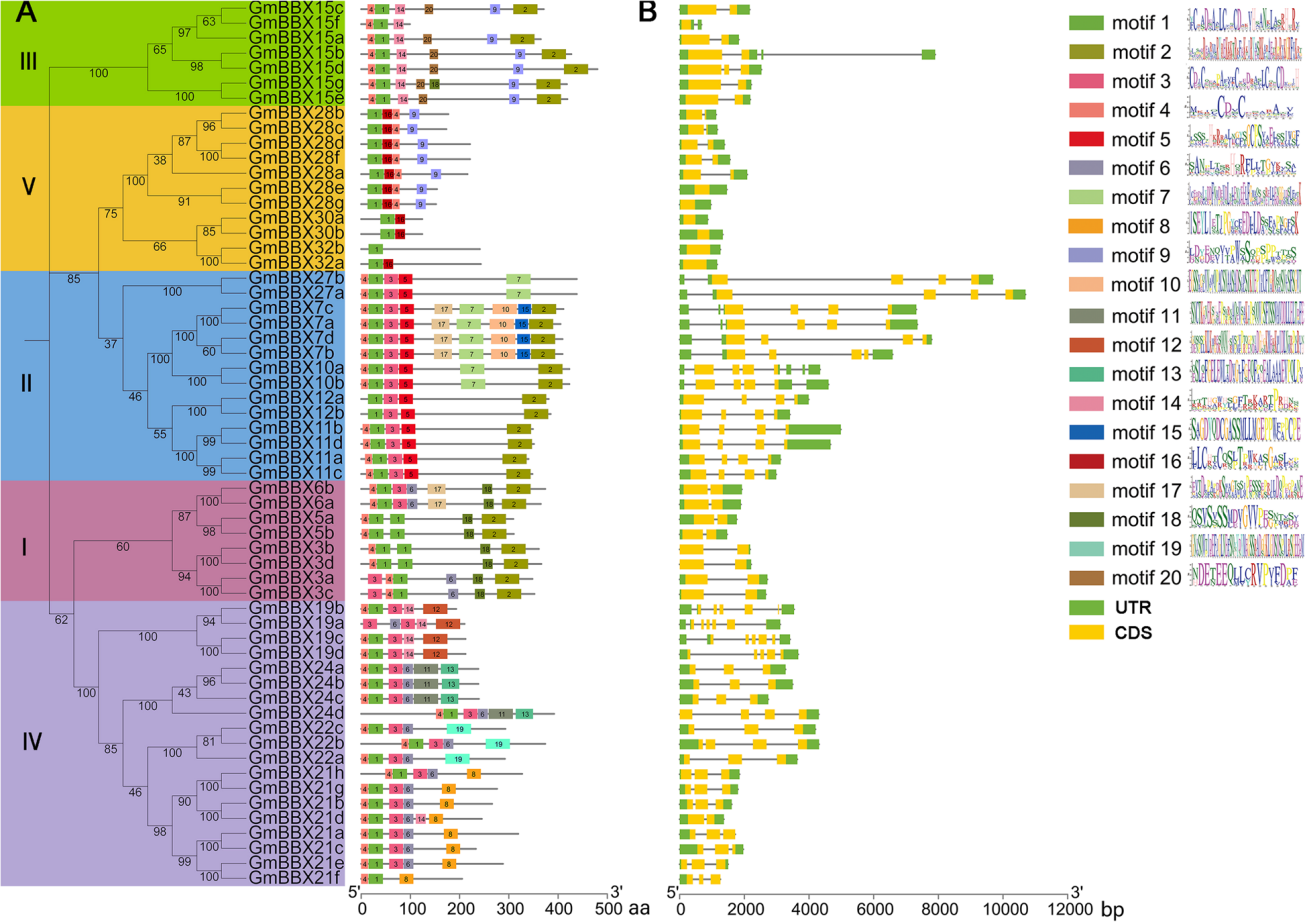
Schematic diagrams for motif compositions of GmBBX proteins and their gene structures. **A** Motif compositions of the 59 GmBBX proteins. The online software, MEME, was used with the maximum number of motifs being set at 20. The left panel represents phylogenetic tree of GmBBXs, and the Roman numerals (I-V) indicate the five groups; the right panel refers to the schematic diagram of motif compositions, and each motif is represented by a number in a colored box; the sequence logos of 20 motifs were shown in the right-most position. **B** Exon-intron structures of *GmBBX* genes in soybean. Exons and UTR are represented by yellow boxes and green boxes, respectively, and grey lines between exons represents introns. The sequence lengths of each protein and gene are represented by grey bars at the bottom

Furthermore, 20 motifs were identified in the 59 GmBBX proteins (Fig. 4A). The motifs 1 and 4 were related to the B-box1, and the motif 3 was corresponded to the B-box2. Besides, the motif 2 was related to CCT domain. It was observed that 59 GmBBX proteins showed diverse motif compositions (Fig. 4A). For example, the motifs 18, 20 and 16 were only present in the clade I, clade III and clade V, respectively; the motifs 10, 12, 8, 19 and 13 were only present in the GmBBX7s, GmBBX19s, GmBBX21s, GmBBX22s and GmBBX24s, respectively (Fig. 4A). Additionally, GmBBX proteins at the same clade in the phylogenetic tree basically showed similar motif compositions (Fig. 4A). These observation implied the functional diversity and redundancy of GmBBXs.

The exon/intron structures of 59 *GmBBX* genes were also constructed according to their coding and genomic sequences. It was observed that *GmBBX* genes showed a variation in the number of exons (Fig. 4B). One gene in the clade III (*GmBBX15f*) and six genes in the clade V (*GmBBX28e*/*28 g*/*30a*/*30b*/*32a*/*32b*) only had one exon, and the other *BBX* genes contained two to six exons (Fig. 4B). Further observation indicated that *GmBBX27a* and *GmBBX15f* were the longest (10.7 Kb) and shortest (677 bp) *BBX* genes with four and one exon(s), respectively (Fig. 4B). Noticeably, six *GmBBX*s contained one exon without intron. *GmBBX* genes at the same clade in the phylogenetic tree basically showed similar exon/intron structures (Fig. 4B). For example, all the *BBX* genes in the clade I and clade II harbored 2 and 4 exons with intron intervals, respectively (Fig. 4B). These observations supported that the *GmBBX* pairs at the same clade might contribute to gene family expansion with less functional diversification.

### GmBBX family might perform functions in response to salt stress

**Fig. 5:**
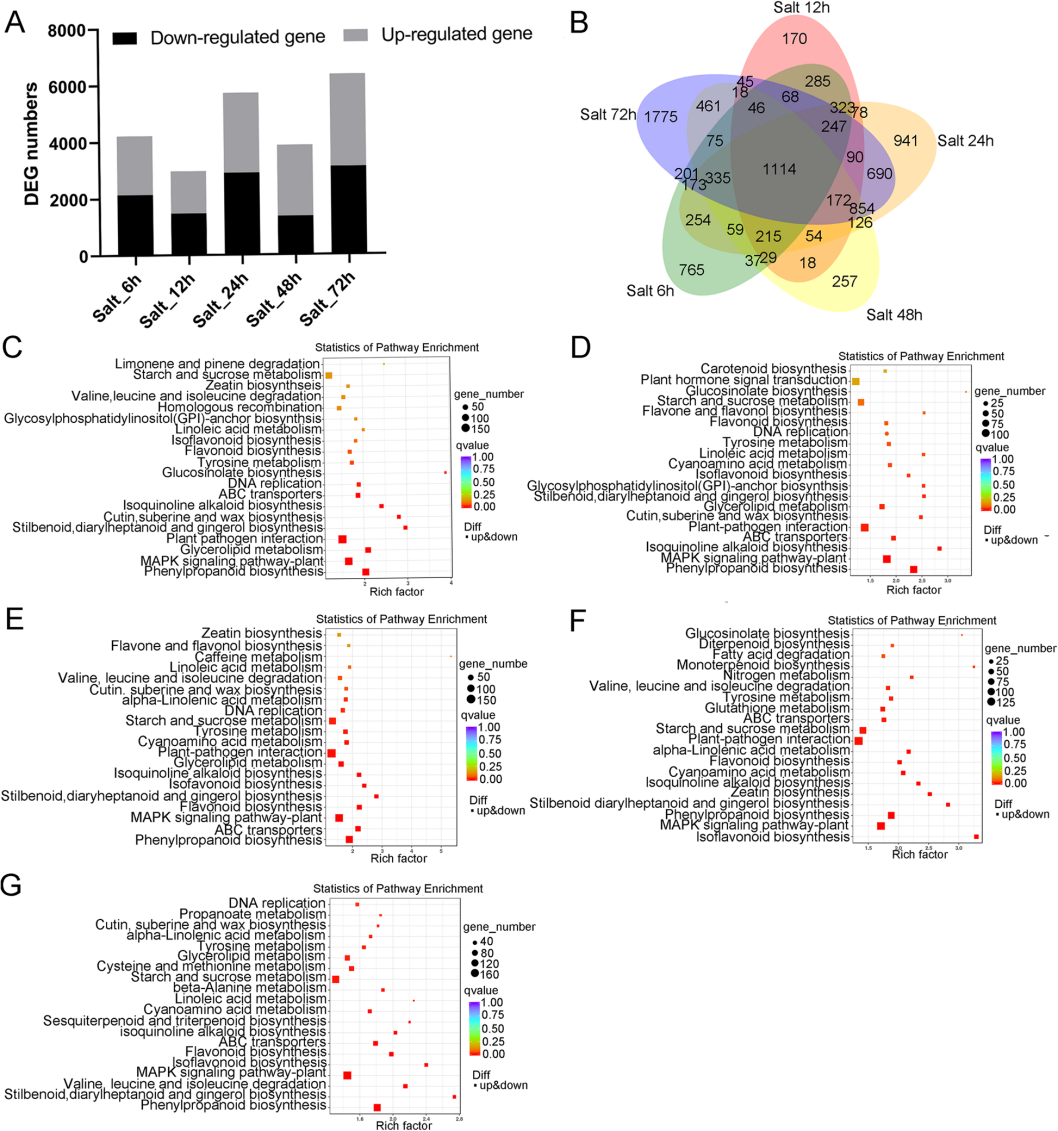
Transcriptomic analysis of soybean seedlings under salt treatment. **A** Numbers and Venn diagram **B** of differentially expressed genes (DEG) under salt treatment for 0 h, 6 h, 12 h, 24 h, 48 h, 72 h as compared to the transcript level at 0 h after salt treatment. (C-G) KEGG enrichment pathway analysis of DEGs under salt treatment for 6 h (**C**), 12 h (**D**), 24 h (**E**), 48 h (**F**), 72 h (**G**). The vertical axis indicates the pathway name, and the horizontal axis shows the Rich factor. The size of the rectangles represents the number of genes in the pathway and the color indicates q-value. KEGG analysis was performed based on the method as described by Kanehisa et al. [34, 35]. Soybean seedlings were exposed to the salt stress of 200 mM NaCl for 0 h, 6 h, 12 h, 24 h, 48 h and 72 h

To investigate transcript profiling under salt stress, we harvested the soybean issues with salt treatment for 0 h, 6 h, 12 h, 24 h, 48 h and 72 h, which were subsequently applied to RNA-seq analysis. To understand dynamic response of soybean genes to salt stress, the altered genes were counted between the salt treatment for 0 h and the other five treatment timepoints. As shown in Fig. 5A-B, 4226, 2972, 5725, 3870 and 6364 genes were transcriptionally up-regulated or down-regulated at 6 h, 12 h, 24 h, 48 h and 72 h after salt treatment, respectively. KEGG analysis indicated that the altered genes after 6 h salt treatment were enriched in MAPK signaling transduction, phenylpropanoid biosynthesis, starch and sucrose metabolism, plant-pathogen interaction (Fig. 5C). After 12 h, 24 h and 48 h salt treatment, the enriched genes were distributed not only in the plant-pathogen interaction pathway, phenylpropanoid biosynthesis pathway, and MAPK signaling pathway, but also in the plant hormone signal transduction pathway (Fig. 5D-F). After 72 h salt treatment, the altered genes were mainly enriched in the starch and sucrose metabolism pathway, phenylpropanoid biosynthesis pathway and MAPK signaling pathway (Fig. 5G).

**Fig. 6:**
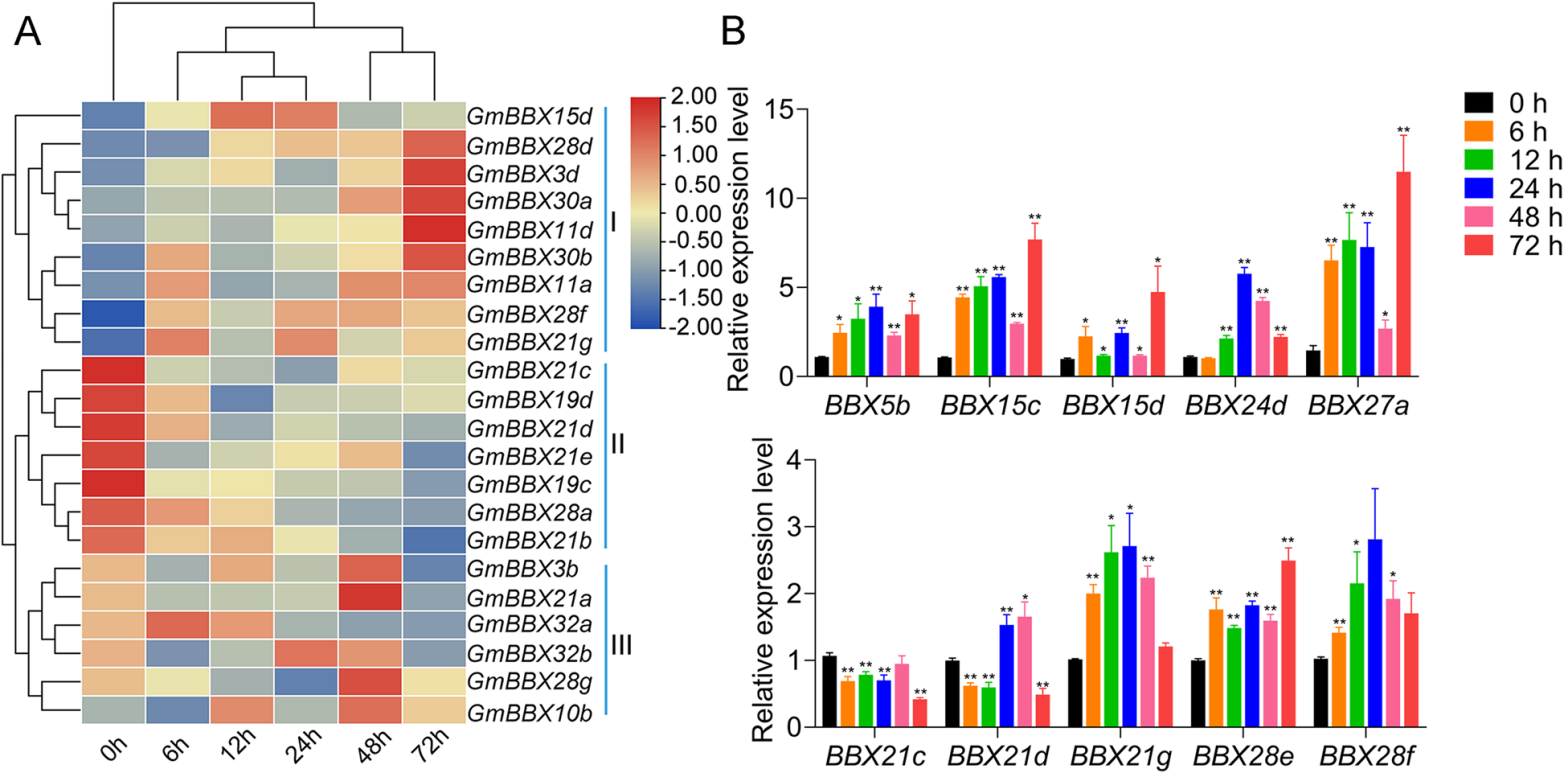
Expression analysis of *GmBBX* genes under salt treatment. **A** Transcript profiling of *GmBBX* genes based on RNA-Seq analysis. The heatmap was generated with the FPKM values of the 22 salt-stress-responsive *GmBBX*s using the online tool, TBtools, and the color scale beside the heat map indicates gene expression levels, low transcript abundance indicated by blue color and high transcript abundance indicated by red color. The 22 salt-stress-responsive *GmBBX*s were classified into three groups Group I, *GmBBX3d/11a/11d/15d/21 g/28d/28f/30a/30b*; Group II, *GmBBX19c/19d/21b/21c/21d/21e/28a*; Group III, *GmBBX3b/10b/21a/28 g/32a/32b*. **B** Gene expression patterns of *GmBBX* genes using qRT-PCR analysis. Soybean seedlings were exposed to the salt stress of 200 mM NaCl for 0, 6, 12, 24, 48 and 72 h, and total RNAs for RNA-Seq and qRT-PCR analysis were extracted from salt-treated seedlings at the six above-mentioned timepoints. The qRT-PCR data were normalized against *GmSUBI3*, and the expression level at the first timepoint (0 h) was set as 1. Error bars indicate SE of three biological and technical replicates, and significant differences are denoted by asterisk(s) (*p* < 0.01 or *p* < 0.05)

To investigate transcript profiling of the 59 *GmBBX* genes under salt stress, we extracted their corresponding transcriptomic data. Consequently, 59 *GmBBX* genes showed different transcript profiling under salt stress (Fig. S4 and Table S5). It was observed that 10 out of 15 *GmBBX*s (*GmBBX3a/3b/5a/5b/15 g/19b/22b/28b/30a/30b*), which were predicted to be distributed in chloroplast, were up-regulated as salt stress time extended (Table S1 and Fig. S4). Among them, 22 *GmBBX* genes were transcriptionally altered with at least two-fold changes by salt stress, which were grouped into 3 categories according to their response to salt stress (Fig. 6A). The category I comprised of 9 genes (*GmBBX3d/11a/11d/15d/21 g/28d/28f/30a/30b*) (Fig. 6A), which were obviously increased in stress duration, especially *GmBBX11d*, *GmBBX28d*, *GmBBX30a* and *GmBBX30b* with 4.36, 5.38, 11.62 and 15.34 fold changes. In the category II, 7 *GmBBX* genes (such as *GmBBX19c/19d/21b/21c/21d/21e/28a*) were clearly decreased as stress time extended (Fig. 6A). Especially, *GmBBX21c* and *GmBBX21d* were reduced to 20.8 and 26.2% of the non-salt-treated control. In the category III, the *GmBBX*s were transcriptionally decreased at the early stress stages and afterward increased, including *GmBBX3b/10b/21a/28 g/32a/32b* (Fig. 6A).

Furthermore, 10 *GmBBX* genes were chosen for qRT-PCR to examine their expression patterns after salt treatment for 0 h, 6 h, 12 h, 24 h, 48 h and 72 h. Consistent with our RNA-Seq data, nine *GmBBX*s were transcriptionally increased by salt stress such as *GmBBX5b*/*15c*/*15d*/*21d/21 g*/*24d*/*27a*/*28e/28f*, while *GmBBX21c* showed decreased expression pattern under salt stress condition (Fig. 6B).

Additionally, the *cis*-acting elements in the promoter regions of *GmBBX* genes were predicted using the online tool, PlantCARE. As shown in Fig. S5, several *cis*-acting elements were observed, including light-responsive elements, stress-responsive elements (Low temperature, Wound, Drought, Defense, Anoxic, Maximal elicitor-mediated activation), hormonal response (Abscisic acid, Salicylic acid, Methyl jasmonate, Gibberellin, Auxin), development-related elements (Meristem, Differentiation, Endosperm, Seed), and other elements (Flavonoid, Circadian, Anaerobic, Zein). Phylogenetic analysis was also performed using the promoter sequences of the 59 *GmBBX* genes. It was showed that the promoters of some homologous genes were clustered at the same clade in the phylogenetic tree. Further observation indicated that the corresponding gene pairs at the same clade showed similar expression patterns and compositions of *cis*-acting elements for example *GmBBX6a* and *GmBBX6b*, *GmBBX7b* and *GmBBX7d*, *GmBBX11b* and *GmBBX11d* (Fig. S4 and Fig. S5). Abscisic acid is involved in plant tolerance to various stresses with inclusion of salt stress. It was observed that 43 *GmBBX*s had one or more abscisic acid -responsive element(s) (Fig. S5).

### Potential transcription factor binding sites in salt-responsive *GmBBX* genes and their protein interactors

**Fig. 7:**
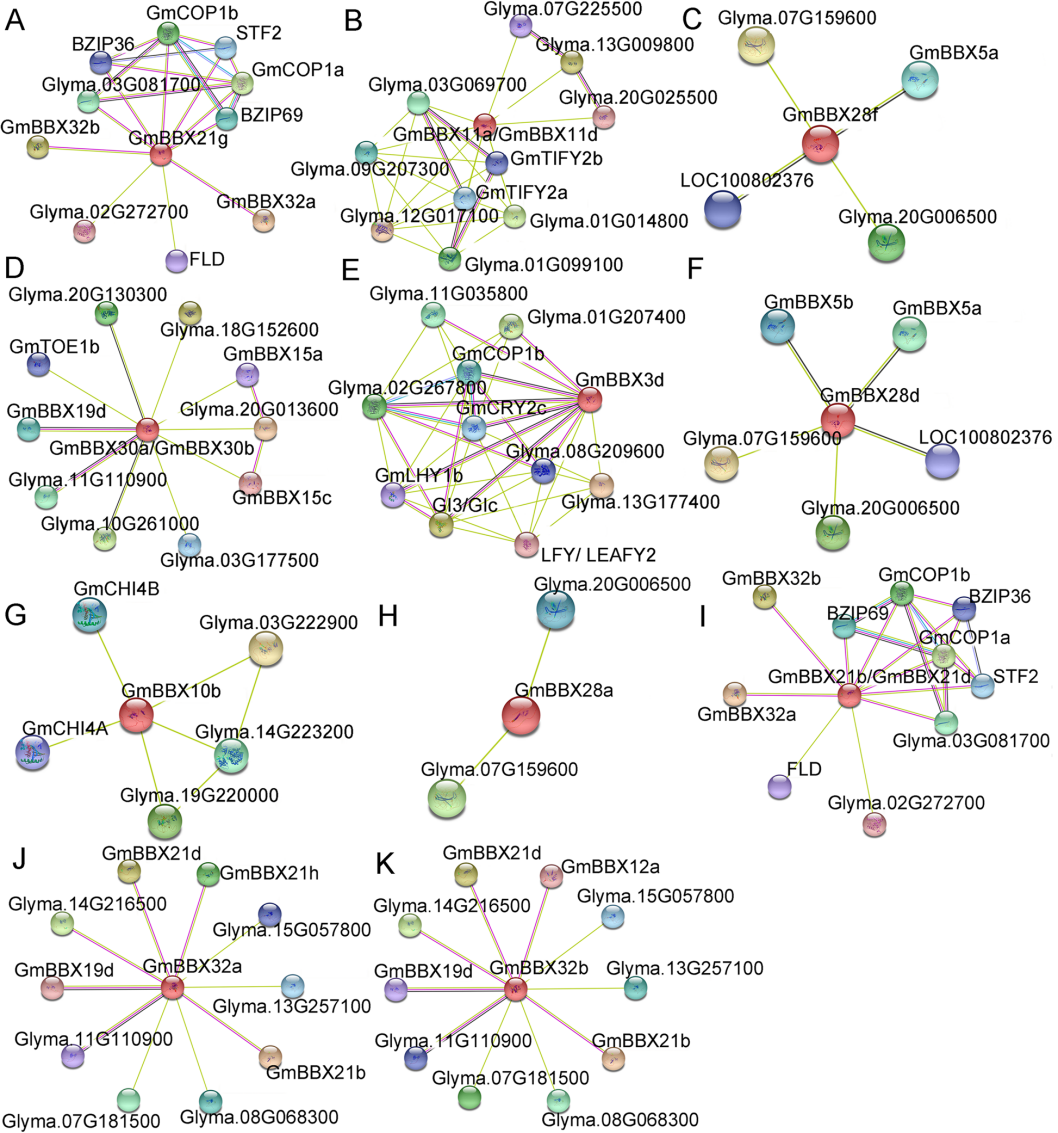
Predicted interactors of the 16 salt-responsive GmBBX proteins using the online program STRING (https://string-db.org/). **A**-**G** Protein-protein interaction of the 7 up-regulated *GmBBX* genes (*GmBBX3d/10b/11a/11d/21 g/28f/28d/30a/30d*) under salt stress. **H**-**K** Protein-protein interaction of the 5 down-regulated *GmBBX* genes (*GmBBX21b//21d/28a/32a/32b*). The red balls indicate the GmBBX proteins, and the other colored balls represent the interactors of the GmBBXs. The interactors include transcription factors (bZIP, TIFY, KAN2, HY5, FLD, AP2-LIKE, CO, LFY, CCA1-LIKE), nuclear proteins (GIGANTEA, Nuclear ribonucleoprotein, Nuclear transport factor), Enzymes (E3 ubiquitin-protein ligase, DNA photolyase, Chalcone isomerase, 4-coumarate--CoA ligase-like), or other proteins (CRY, Secretory protein, Chaperone, Plectin)

To provide the clues regarding the interaction of GmBBXs with other factors in response to salt stress, 16 GmBBXs were chosen to predict their interactors using the online program, STRING, against the soybean protein database (https://string-db.org/), including 9 up-regulated (*GmBBX3d/10b/11a/11d/21 g/28f/28d/30a/30d*) and 7 down-regulated (*GmBBX21a/21b/21c/21d/28a/32a/32b*) *GmBBX*s with at least 2-fold changes relative to non-salt control. Consequently, all the GmBBXs but GmBBX21a/21c showed interaction with other proteins such as transcription factors (bZIP, TIFY, KAN2, HY5, FLD, AP2-LIKE, CO, LFY, CCA1-LIKE), nuclear proteins (GIGANTEA, Nuclear ribonucleoprotein, Nuclear transport factor), Enzymes (E3 Ubiquitin-protein ligase, DNA photolyase, Chalcone isomerase, 4-coumarate--CoA ligase-like), and other proteins (CRY, Secretory protein, Chaperone, Plectin) (Fig. 7 and Table S6). Further observation showed that the up-regulated and down-regulated BBXs shared some interactors in common such as GmCOP1a, GmCOP1b, GmFLD, whereas different interactors were observed between up-regulated and down-regulated GmBBXs for example GmBBX30a/30b-GmTOE1b, GmBBX3d-GmGI3/GmCRY, GmBBX10b-GmCHI in the up-regulated group (Fig. 7A-G); GmBBX32a/32b-GmBBX21b/21d, GmBBX21d/21b-GmSTF2 in the down-regulated group (Fig. 7H-K). Noticeably, the interactions between GmBBXs were observed for example GmBBX21g-GmBBX32a/32b, GmBBX30a/30b-GmBBX15a/15c/19d, GmBBX28f-GmBBX5a, GmBBX28d-GmBBX5a/5b, GmBBX32a-GmBBX19d/21b/21d/21 h, GmBBX32b-GmBBX12a/19d/21b/21d (Fig. 7 and Table S6).

**Fig. 8:**
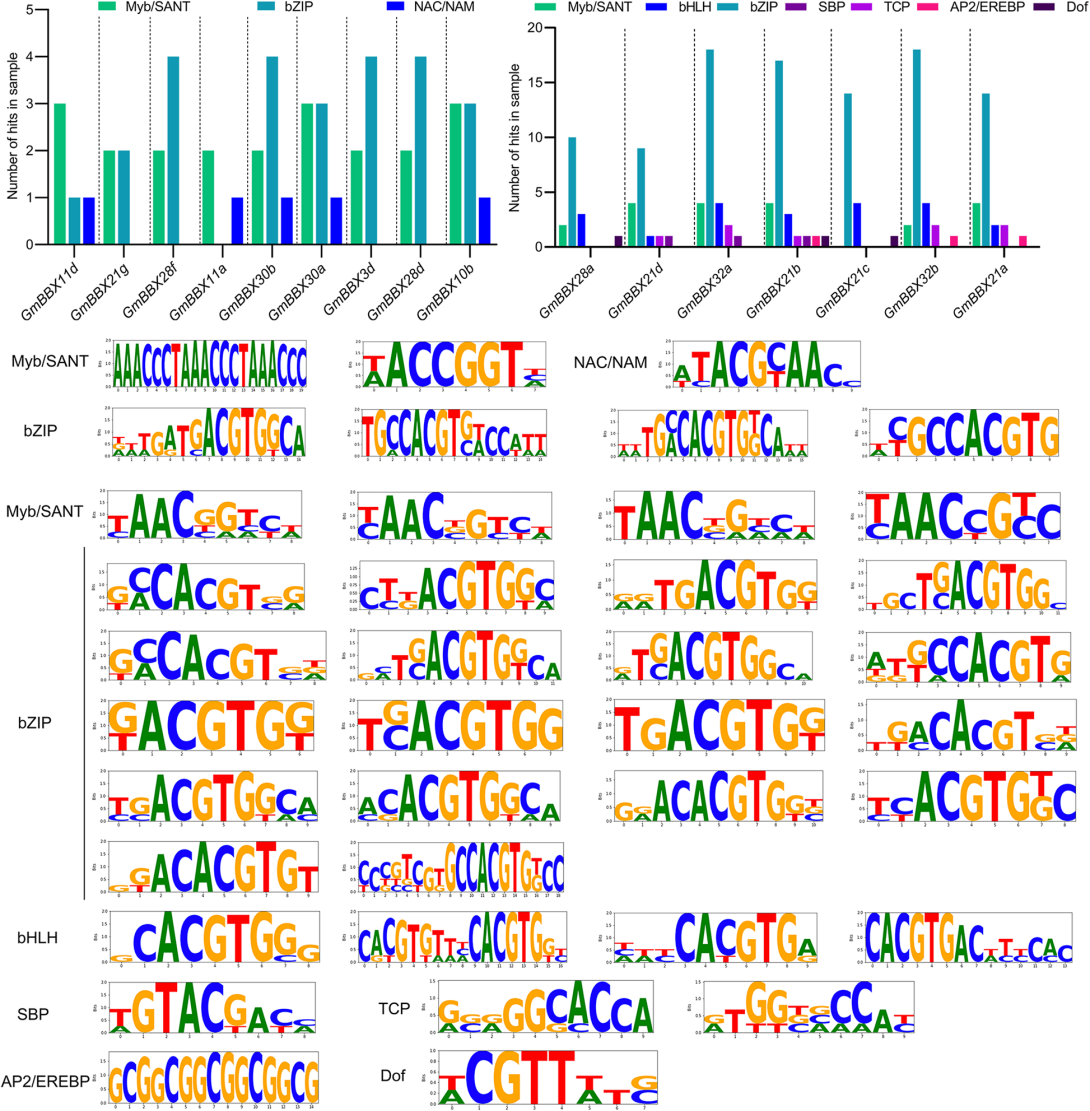
The predictions of transcription factors binding to the 16 salt-responsive *GmBBX* genes. **A** The potential transcription factors binding to the 9 up-regulated *GmBBX*s (*GmBBX3d/11a/11d/15d/21 g/28d/28f/30a/30b*) under salt stress such as MYB/SANT, bZIP, NAC/NAM. **B** The potential transcription factors binding to 7 down-regulated *GmBBX* genes (*GmBBX21a/21b/21c/21d/28a/32a/32b*) under salt stress such as MYB/SANT, bZIP, NAC/NAM, bHLH, SBP, TCP, AP2/EREBP, and Dof. The prediction was performed using the online program, TDTHub (http://acrab.cnb.csic.es/TDTHub/). **C**-**D** The logos of the binding sites for the up-regulated (**C**) and down-regulated (**D**) *GmBBX* genes. The X axis in the logos represents the position of each amino acid, and the Y axis and the height of each letter represent the degree of conservation of each residue in all proteins

To provide the hints regarding transcription factors involved in the regulation of the salt-responsive *GmBBX*s, potential binding sites at the promoter regions of the 16 *GmBBX* gene were predicted using the online tool, TDTHub. As shown in Fig. 8, all the salt-responsive *GmBBX* genes were bound by bZIP and MYB transcription factors except *GmBBX21c*. Further observation indicated that the binding sites were different between the salt-induced and salt-suppressed *GmBBX* genes. In brief, all the 9 salt-induced *GmBBX* genes were possibly bound by the transcription factors, MYB/SANT, bZIP and NAC/NAM (Fig. 8A and C). For example, four genes (*GmBBX10b/11d/30a/30b*) harbored the binding sites of MYB/SANT, bZIP and NAC/NAM (Fig. 8A and C). The binding sites of MYB/SANT and bZIP were observed in the promoter regions of the four *GmBBX* genes such as *GmBBX3d/21 g/28d/28f*, while *GmBBX11a* possessed the binding sites of MYB/SANT and NAC/NAM (Fig. 8A and C). In contrast, seven types of transcription factors were predicted for the salt-suppressed *GmBBX* genes, including MYB/SANT, bHLH, bZIP, SBP, TCP, AP2/EREBP and Dof (Fig. 8B and D). For example, *GmBBX21b* harbored the binding sites of all the seven types of transcription factors. Five binding sites were observed at the promoter regions of *GmBBX21d/32a* (MYB/SANT, bHLH, bZIP, SBP and TCP), and *GmBBX21a/32b* (MYB/SANT, bHLH, bZIP, TCP and AP2/EREBP) (Fig. 8B and D). *GmBBX28a* and *GmBBX21c* were shown to harbor the binding sites of four (MYB/SANT, bHLH, bZIP and Dof) and three (bHLH, bZIP and Dof) types of transcription factors, respectively (Fig. 8B and D).

## Discussion

*BBX* genes are key regulators with the involvement of mediating developmental processes and stress responses, which have been identified and functionally characterized in many plant species [2–12]. However, our understanding of BBX family is greatly limited in soybean. In this study, the members of soybean BBX family were comprehensively analyzed in diverse aspects.

We totally identified 59 *BBX* genes in soybean (Table S1), and the number of *GmBBX* genes was significantly more than the ones in Arabidopsis (32) [3], rice (30) [5], and tomato (29) [7]. The genome size of soybean (Williams 82) is approximately 1115 Mb [36, 37], and 52,872 genes have been predicted in the Wm82v4 assembly, which is roughly 1.93 times as many genes as annotated in Arabidopsis (27,411, TAIR10). Thus, it is reasonable for soybean to have a large number of *BBX* genes. The genome size as well as gene family members in plants have been influenced by evolutionary events such as duplication and polyploidy events [38, 39]. Accordingly, it seems that BBX family genes have been more subjected and extended in soybean. Phylogenetic analysis indicated that GmBBXs can be divided into five clades and clustered together with the BBXs from the other plant species (Fig. 1), suggesting that they might have undergone similar evolutionary diversification. Previous studies showed that the *BBX* genes in the same clade might perform similar functions. For example, the Arabidopsis *BBX* genes in the clade I (such as *AtCO* and *AtCOL*) were mostly associated with photoperiod or photoperiod-regulated flowering [13–15], while the majority of the *BBX* genes in the clade IV was related to the regulation of light signal in plants, including *AtBBX18* (*DBB1a*), *AtBBX19* (*DBB1b*), At*BBX24* (*STO*), *AtBBX25* (*STH1*), *AtBBX21* (*STH2*) and *AtBBX22* [16–19]. Similarly, three tomato BBXs (SlBBX7 and SlBBX9 in the clade II; SlBBX17 in the clade V) might play important roles in response to cold or heat stress [40, 41], while three SlBBXs (SlBBX19 and SlBBX20 in the clade IV; SlBBX26 in the clade V) were possibly involved in the regulation of fruit ripening [32, 42]. Thus, the function-known homologs of *GmBBX* genes in other plant species provided clues for further studying their corresponding functions. Based on the type and number of functional domains (for example B-box and CCT), GmBBX family was divided into five subfamilies (Clade I-V), indicating that functional diversity existed in soybean BBX family (Fig. 3). It was observed that the numbers of different subfamilies varied in different plant species. In Arabidopsis, for example, 13, 4, 8 and 7 *BBX* genes were distributed in the group I/II, group III, group IV and group V, respectively [3]; 7, 10, 10 and 3 in rice, respectively [5]; 20, 6, 20 and 13 in soybean, respectively. These results suggested that although BBX family in different species might have a common ancestor, their subsequent evolutionary processes were relatively independent.

It has been generally accepted that tandem and segmental duplications of chromosomal regions were main contributors for gene expansion during evolution [33]. In this study, 38 duplication sets covering 56 *GmBBX* genes were not only clustered into a discrete clade in phylogenetic tree with high protein identity (For example ten pairs of *GmBBX*s showing the identity of 90.10–95.24%) and similar motif compositions (Fig. 4A and Table S3), but also exhibited low Ka/Ks ratios (0.048–0.469) (Table S2). These observations suggested that each pair of duplicated genes possibly had the closest evolutionary relationship and shared similar functions in soybean. Previous studies have reported that exon-intron structures can be used to support phylogenetic relationship in gene family [43]. Intriguingly, the *GmBBX* genes at the same clade in the phylogenetic tree basically showed similar exon/intron structures (Fig. 4B), supporting that they might be generated through segmental duplication. It is not surprising since soybean had undergone whole-genome duplication event, which led to duplication of at least 75% of genes in soybean genome [44, 45]. However, no tandem duplication event was observed for the expansion of BBX family in soybean. These observations suggested that segmental duplication was primarily responsible for the expansion of BBX family during evolution in soybean. Low exon number was observed into BBX gene structure. It has been stated that genes with fewer exons are classified as early response genes and are induced faster [46, 47].

It is well known that salt stress can lead to severely limited growth and significant reduction of crop productivity [29]. Increasing evidence indicated that *BBX* genes are involved in plant response to salt stress. For instance, overexpression of *AtSTO* (*AtBBX24*) promoted root growth at high salinity in Arabidopsis [24], while *MdBBX1* transgenic plants showed higher survival rate under salt stress [28]. In this study, we provided three aspects of information to support that *GmBBX*s might be important regulators to respond to salt stress in soybean. Firstly, our RNA-seq data indicated that 22 *GmBBX* genes were transcriptionally altered with more than two-fold changes by salt stress (Fig. 6A and Fig. S4), which is consistent with previous reports that four *BBX* genes in grape and five *BBX* genes in rice were up-regulated under salt stress [12, 22], while *BrBBX15*, *BrBBX17* and *BrBBX6* were clearly induced by NaCl in *Brassica rapa* [48]. The altered expression patterns of *GmBBX* genes suggested their functional roles in response to salt stress. Secondly, the transcription factors such as bZIP, NAC/NAM, MYB were predicted to bind to the salt-responsive *GmBBX* genes (Fig. 8 and Table S7). It was reported that a number of transcription factors, like bZIP, NAC/NAM, MYB, are involved in the regulation of salt tolerance in soybean. For example, *GmbZIP15*, *GmbZIP2* and *GmbZIP19* were positively or negatively implicated in response to salt stress in soybean, respectively [49–51]; the overexpressions of *GmNAC06*, *GmNAC11*, *GmNAC20*, *GmNAC109* and *GmNAC181* enhanced salt tolerance in soybean or Arabidopsis [52–56]; *GmMYB84*, *GmMYB76* and *GmMYB177* conferred soybean tolerance to salt stress [57]. Additionally, interactors of GmBBX proteins supported their functional roles in response to salt stress such as GmGI, GmTOE1b, GmCOP1, GmCHI (Fig. 7 and Table S6). It was reported that the suppression of *GmGI*, *AtGI*, *OsGI*, *BrGI*, *PagGI*s conferred salt tolerance in soybean, Arabidopsis, rice, *Brassica rapa* and poplar [58–62]; the IbBBX24-IbTOE3-IbPRX17 regulatory module in sweet potato facilitated plant tolerance to salt stress [27]; the *cop1* mutants showed more tolerant to salt stress as compared with WT in Arabidopsis [63]; the salt tolerance of composite soybean plants and transgenic Arabidopsis was negatively regulated by *AtCHI* [64, 65]. Thus, we speculated that *GmBBX* genes might be important regulators in response to salt stress in soybean.

## Conclusions

In this study, 59 *GmBBX* genes were identified and characterized in soybean, including phylogenetic relationship, chromosomal localization, gene duplication, gene structure, motif composition, conserved domain, and gene expression pattern under salt stress. Furthermore, salt-responsive *GmBBX*s were computationally investigated for their interactors and transcriptional regulators. These findings will contribute to future research in regard to the functions and regulatory mechanisms of soybean *BBX* genes in response to salt stress.

## Materials and methods

### Identification and annotation of *BBX* genes in soybean

To identify the *BBX* genes in soybean, the soybean reference genome assembly (*Glycine max* Wm82.a2.v1) and the gene annotation file were downloaded from the Phytozome13 (https://phytozome-next.jgi.doe.gov/) [66] and Ensembl Plants database (http://plants.ensembl.org/info/data/ftp/index.html) [67], respectively. The Hidden Markov Model (HMM) profile for the B-box-type zinc finger domain (PF00643) was obtained from the Pfam (http://pfam.xfam.org/) [68] and used to identify *BBX* genes in soybean by the Simple HMM Search of TBtools [69]. Furthermore, the domains of BBX proteins were checked by the two online programs, SMART (http://smart.embl-heidelberg.de/smart/set_mode.cgi?NORMAL=1) and CDD (https://www.ncbi.nlm.nih.gov/Structure/cdd/wrpsb.cgi). The CDS sequences and protein sequences of soybean BBXs were downloaded from Phytozome13. The molecular weight (MW) and isoelectric point (pI) of soybean BBX proteins were calculated using the resource portal, ExPASy (https://web.expasy.org/compute_pi/) [70]. The subcellular localization of each GmBBX protein was predicted using the online software, WoLF PSORT (https://www.genscript.com/wolf-psort.html?src=leftbar).

### Phylogenetic and conserved domain alignments analysis

Amino acid sequences of the B-box and CCT domains were aligned using MEGA11 [71] and DNAMAN. The protein homology analysis was calculated using the online tool, MUSCLE of EMBL-EBI (https://www.ebi.ac.uk/Tools/msa/muscle/). The sequence logos were created using WebLogo (http://weblogo.berkeley.edu/logo.cgi). Multiple sequence alignments of GmBBXs were performed using MEGA11 and DNAMAN. The phylogenetic trees were generated by the maximum likelihood method with 1000 bootstrap replications with MEGA11 [71]. The information of BBXs in Arabidopsis, tomato, maize and rice were downloaded from the Phytozome13 or TAIR (https://www.arabidopsis.org/), respectively.

### Analysis of exon-intron structures and conserved motifs

Exon-intron structures of *BBX* genes in soybean were determined by the coding sequence and the genomic sequence in the *Glycine max* Wm82.a2.v1. The diagrams of exon-intron structures were generated by the Gene Structure View (Advanced) of TBtools [69]. The conserved motifs of GmBBX proteins were identified using the online software, MEME (https://meme-suite.org/meme/tools/meme) [72], with the maximum number of motifs being set at 20, and the map of motifs was constructed by TBtools [69].

### Chromosomal localization and synteny analysis

The chromosomal localization of each *GmBBX* gene was identified according to the physical location from the *Glycine max* Wm82.a2.v1 genome annotation. Synteny analysis of the *BBX*s within soybean as well as between soybean and Arabidopsis was conducted using the One Step MCScanX of TBtools [69]. Synteny analysis and chromosomal location diagrams were generated by the program, Circos-0.69-9 (http://circos.ca) [73]. The nonsynonymous (Ka) and synonymous (Ks) substitution rate of each *BBX* gene pairs was calculated by the Simple Ka/Ks Caculator of TBtools [69]. The divergence time of each gene pairs was calculated with the Ks values via the follow formula: T = Ks/2λ (λ = 6.1 × 10 ^− 9^ for soybean) [74].

### Promoter analysis of *GmBBX* genes

One thousand five hundred bp interval upstream of the translation initiation site of each *GmBBX* gene was considered as promoter region and applied to the online program, PlantCARE (http://bioinformatics.psb.ugent.be/webtools/plantcare/html/), for promoter analysis. The corresponding data were followed by a visualization using the online tool, TBtools [69]. The phylogenetic trees were generated using the promoter sequences of *GmBBX*s by the maximum likelihood method with MEGA11 [71].

### Plant materials and salt stress treatment

Salt stress of ten-day-old soybean seedlings (Williams 82) were performed as previously described [75]. Briefly, soybean seeds were sterilized and germinated in plate with wet filter paper. Subsequently, four well-germinated seeds were selected and sown on each pot filled with 65 g vermiculite. All the seedlings were grown under a 14 h/10 h (light/dark) photoperiod at 25 °C/20 °C (light/dark) and regularly watered with Hoagland liquid medium. Six pots of ten-day-old seedlings were subjected to salt treatment with the supplement of sufficient 200 mM NaCl solution (200 ml). The ground-above tissues were collected at 0, 6, 12, 24, 48 and 72 h after salt application, respectively. The harvested samples were frozen in liquid nitrogen and stored at − 80 °C for the following RNA-Seq and qRT-PCR. Each treatment timepoint had at least six pots of seedlings, and three biological replicates were performed for each treatment timepoint.

### RNA-Seq analysis

The ground-above tissues at 0, 6, 12, 24, 48 and 72 h after salt application were collected and applied to RNA-Seq analysis. The workflow includes sample preparation, library construction, library quality control and sequencing on Illumina sequencing platform. The raw data was first filtered to get Clean Data. HISAT2 was used to map RNA-seq data reads [76]. StringTie was applied to assemble the mapped reads [77], and DESeq2 was used for differential expression analysis among sample groups [78]. Subsequently, the transcript profiling data of *GmBBX* genes were extracted from RNA-seq data, and the heatmap was generated with the corresponding FPKM values using the online programme, TBtools [69].

### qRT-PCR analysis

The total RNA was exacted via RNAprep Pure Plant Plus Kit (TIANGEN, China). The first-strand cDNA was generated by StarScript II First-strand cDNA Synthesis Mix With gDNA Remover Kit (GenStar, China). qRT-PCR was performed using the Bio-Rad CFX Connect Real-Time PCR Detection System with the reagent of 2 × RealStar Green Fast Mixture (GenStar, China). *GmUBIQUITIN-3* (*GmSUBI3*) was used as the internal reference. The data was analyzed using the Bio-Rad CFX Manager. Three biological replicates with three techniques were conducted for each sample. Primer information is listed in Table S8. Statistical significance of the data was analyzed using independent-samples t-test. Error bars indicate SE and *p*-value < 0.05 (*) or < 0.01 (**).

### Prediction of the binding of transcription factor and protein-protein interaction

The online program, TFBS-Discovery Tool Hub (http://acrab.cnb.csic.es/TDTHub/), [79] was used to predict the transcription factor binding sites (TFBS) of 16 salt-responsive *BBX* genes. The promoter region (3 kb upstream of Translation Initiation Codon) of each *BBX* gene was applied to query TFBS using the general-purpose tool, FIMO, and the minimum s-score threshold was 1%. The diagrams were drawn based on the number of the predicted transcription factors that hit for each *BBX* gene using the TDTHub. Protein-protein interaction was predicted against the databases of *Glycine max* using the online program, STRING (https://string-db.org/).

## Supplementary Information

**Additional file 1:** **Fig. S1.** Distribution and synteny analysis of *BBX* genes on soybean and Arabidopsis chromosomes. The positions on the chromosome of the *BBX* genes from soybean and Arabidopsis are shown on the outside. Colored lines connecting genes syntenic occurrences between *GmBBX*s and *AtBBX*s. 59 soybean BBXs and 32 Arabidopsis BBXs were obtained from Phytozome13 and TAIR, respectively. BBXs 54 orthologous *BBX* gene pairs were observed between the two species, comprising 41 *GmBBX*s and 17 *AtBBX*s. **Fig. S2.** Multiple sequence alignment of GmBBX protein sequences in the clade I and clade II. Protein homology ≥ 33% is shown as yellow, ≥ 50% as blue, ≥ 75% as pink, and 100% as black. The conserved B-box1 domains are marked with green box, the conserved B-box2 domains with red box, the conserved CCT domain with pink box, the VP-motif with blue box, and the conserved amino acid sequence (SANPLASR) with purple box. **Fig. S3.** Alignments and sequence logos of the conserved domains of GmBBX proteins. The domain B-box1 is shown in (A), B-box2 in (B), and CCT in (C). Protein homology ≥ 33% is shown as yellow, ≥ 50% as blue, ≥ 75% as pink, and 100% as black. The X axis in the logos represents the position of each amino acid, and the Y axis and the height of each letter represent the degree of conservation of each residue in all proteins. **Fig. S4.** The heatmap of the 59 *GmBBX* genes under salt stress using the online tool TBtools. Soybean seedlings were exposed to the salt stress of 200 mM NaCl for 0, 6, 12, 24, 48 and 72 h. The heatmap was generated with the FPKM values of the 59 salt- stress-responsive *GmBBX*s using the online tool, TBtools. The color scale beside the heat map indicates gene expression levels, low transcript abundance indicated by green color and high transcript abundance indicated by red color. **Fig. S5.** The *cis*-acting elements in the promoter regions of the 59 *GmBBX* genes. 1,500 bp interval upstream of the translation initiation site of each *GmBBX* gene was considered as promoter region. The phylogenetic tree was generated using the promoter sequences of *GmBBX* genes (the left panel). Fifty-nine promoter sequences were applied for the prediction of *cis*-acting elements using the online program, PlantCARE. The colored boxes in the middle panel represent different *cis*-acting elements, and the sequence length of each promoter is represented by grey bar at the bottom. The symbols in the right panel are corresponding to the colored boxes.

**Additional file 2:** **Table S1.** Information of GmBBX family members in soybean. **Table S2**. Segmental duplications of BBX genes in soybean and KaKs ratios analysis. **Table S3.** Identity between soybean BBX proteins. **Table S4.** Segmental duplications of BBX genes between soybean and Arabidopsis and KaKs ratios analysis. **Table S5**. Information regarding the transcript profiling of GmBBX genes under salt stress (RNA-Seq). **Table S6.** Interactors of the salt-responsive GmBBX proteins. **Table S7.** The information of putative transcription factors potentially binding to GmBBX genes using TDTHub. **Table S8.** Primers used in the study.

## Data Availability

The raw sequencing data from this study has been deposited in the Genome Sequence Archive in BIG Data Center (https://bigd.big.ac.cn/), Beijing Institute of Genomics (BIG), Chinese Academy of Sciences, under the accession number: CRA007841(salt 0 h: CRX484978, CRX484979, CRX484980; salt 6 h: CRX484981, CRX484982, CRX484983; salt 12 h: CRX484984, CRX484985, CRX484986; salt 24 h: CRX484987, CRX484988, CRX484989; salt 48 h: CRX484990, CRX484991, CRX484992; salt 72 h: CRX484993, CRX484994, CRX484995). All data generated or analysed during this study are included in this published article [and its supplementary information files].
