# Supplementary material for: Genome-wide identification of BBX gene family and their expression patterns under salt stress in soybean
Source: BMC Genomics. 2022 Dec 12;23:820. doi: 10.1186/s12864-022-09068-5 (PMC9743715; doi:10.1186/s12864-022-09068-5)
Supplement: Supplementary file 1 — Additional file 1: Fig. S1. Distribution and synteny analysis of BBX genes on soybean and Arabidopsis chromosomes. The positions on the chromosome of the BBX genes from soybean and Arabidopsis are shown on the outside. Colored lines connecting genes syntenic occurrences between GmBBXs and AtBBXs. 59 soybean BBXs and 32 Arabidopsis BBXs were obtained from Phytozome13 and TAIR, respectively. BBXs 54 orthologous BBX gene pairs were observed between the two species, comprising 41 GmBBXs and 17 AtBBXs. Fig. S2. Multiple sequence alignment of GmBBX protein sequences in the clade I and clade II. Protein homology ≥ 33% is shown as yellow, ≥ 50% as blue, ≥ 75% as pink, and 100% as black. The conserved B-box1 domains are marked with green box, the conserved B-box2 domains with red box, the conserved CCT domain with pink box, the VP-motif with blue box, and the conserved amino acid sequence (SANPLASR) with purple box. Fig. S3. Alignments and sequence logos of the conserved domains of GmBBX proteins. The domain B-box1 is shown in (A), B-box2 in (B), and CCT in (C). Protein homology ≥ 33% is shown as yellow, ≥ 50% as blue, ≥ 75% as pink, and 100% as black. The X axis in the logos represents the position of each amino acid, and the Y axis and the height of each letter represent the degree of conservation of each residue in all proteins. Fig. S4. The heatmap of the 59 GmBBX genes under salt stress using the online tool TBtools. Soybean seedlings were exposed to the salt stress of 200 mM NaCl for 0, 6, 12, 24, 48 and 72 h. The heatmap was generated with the FPKM values of the 59 salt- stress-responsive GmBBXs using the online tool, TBtools. The color scale beside the heat map indicates gene expression levels, low transcript abundance indicated by green color and high transcript abundance indicated by red color. Fig. S5. The cis-acting elements in the promoter regions of the 59 GmBBX genes. 1,500 bp interval upstream of the translation initiation site of each GmBBX gene was consider [file 12864_2022_9068_MOESM1_ESM.pdf]

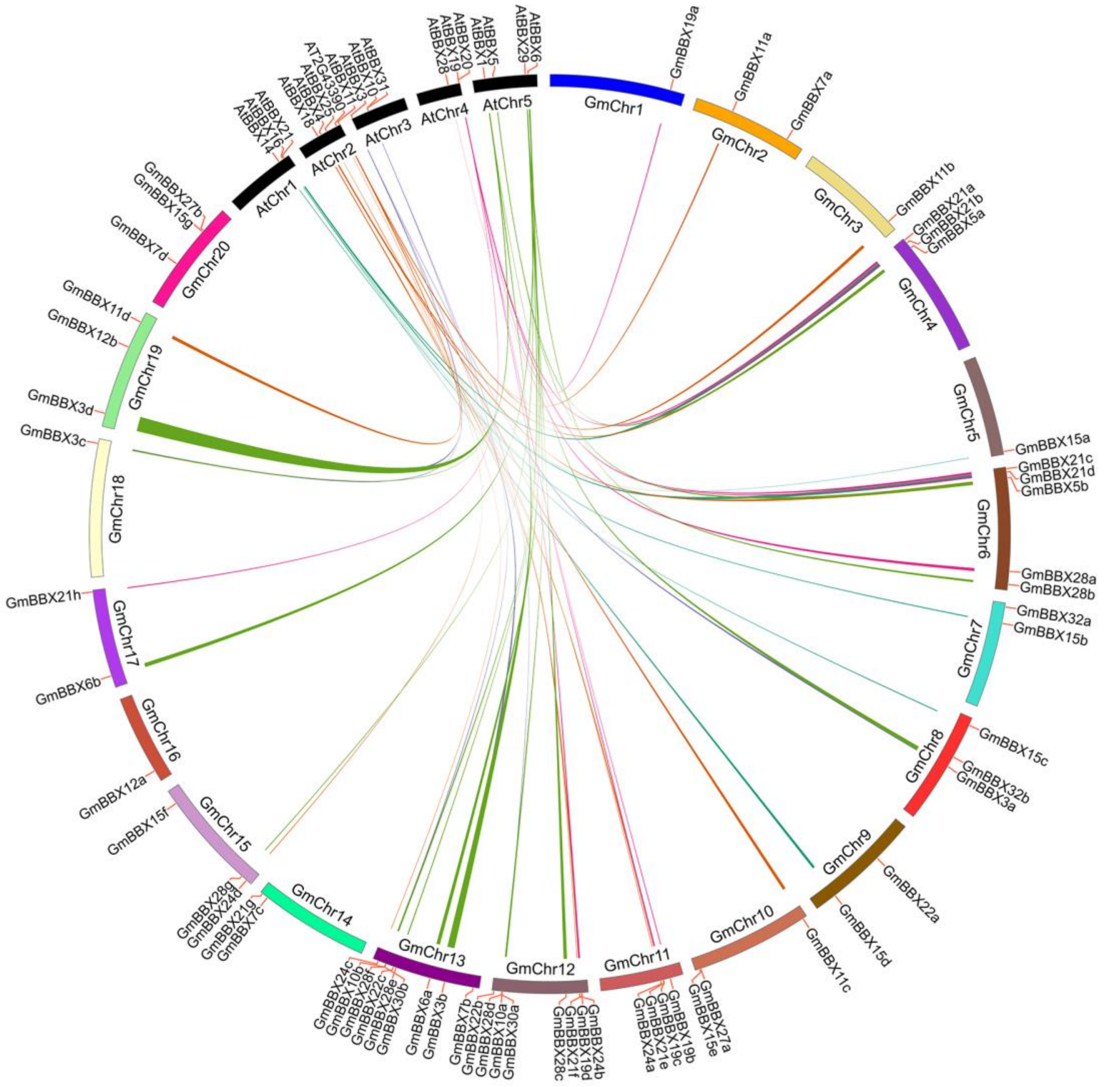

Fig. S1 Distribution and synteny analysis of *BBX* genes on soybean and Arabidopsis chromosomes. The positions on the chromosome of the *BBX* genes from soybean and Arabidopsis are shown on the outside. Colored lines connecting genes syntenic occurrences between *GmBBX*s and *AtBBX*s. 59 soybean BBXs and 32 Arabidopsis BBXs were obtained from Phytozome13 and TAIR, respectively. BBXs 54 orthologous *BBX* gene pairs were observed between the two species, comprising 41 *GmBBX*s and 17 *AtBBX*s.

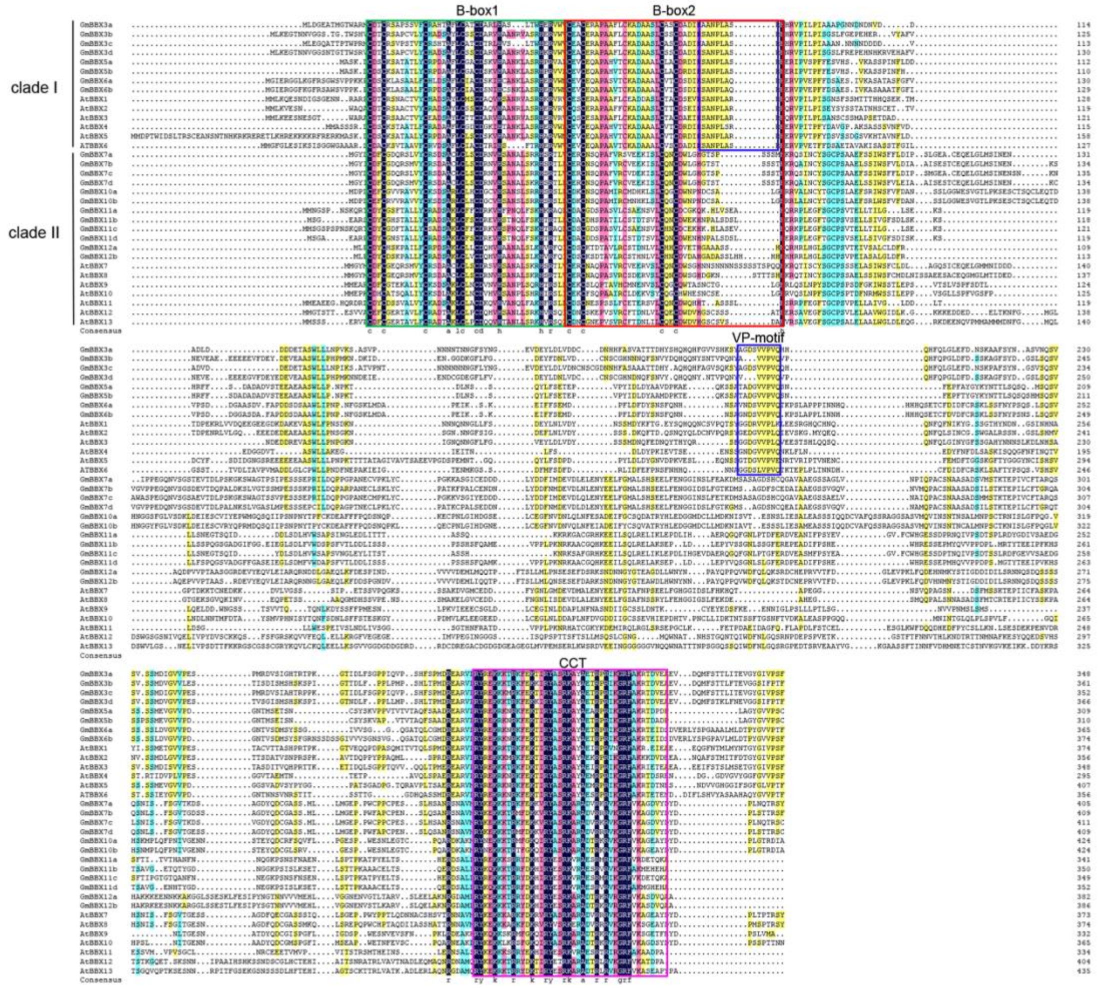

Fig. S2 Multiple sequence alignment of GmBBX protein sequences in the clade I and clade II. Protein homology ≥ 33% is shown as yellow, ≥ 50% as blue, ≥ 75% as pink, and 100% as black. The conserved B-box1 domains are marked with green box, the conserved B-box2 domains with red box, the conserved CCT domain with pink box, the VP-motif with blue box, and the conserved amino acid sequence (SANPLASR) with purple box.

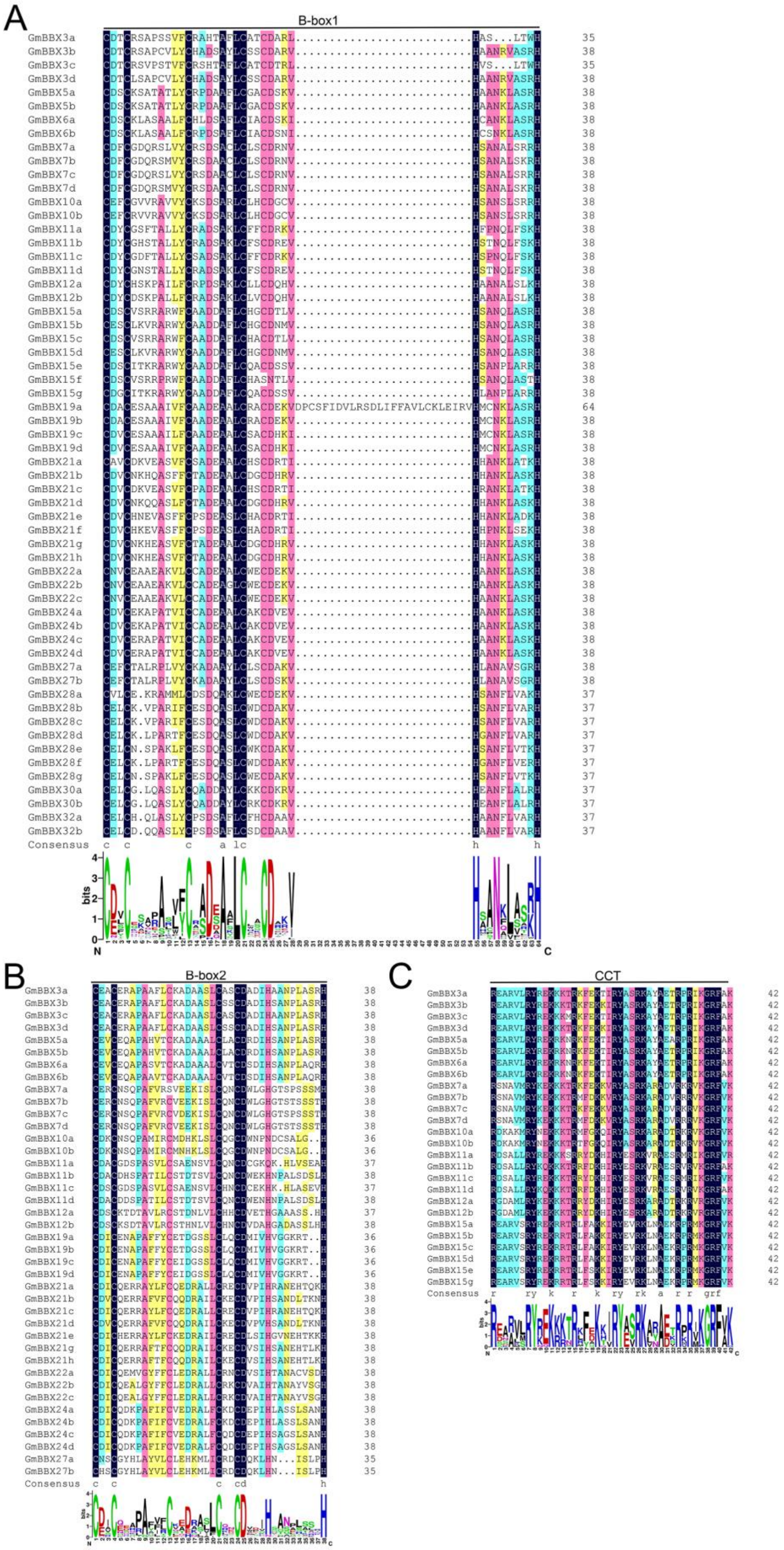
A
B-box1
B
B-box2
C
CCT

Fig. S3 Alignments and sequence logos of the conserved domains of GmBBX proteins. The domain B-box1 is shown in (A), B-box2 in (B), and CCT in (C). Protein homology $\geq$ 33% is shown as yellow, $\geq$ 50% as blue, $\geq$ 75% as pink, and 100% as black. The X axis in the logos represents the position of each amino acid, and the Y axis and the height of each letter represent the degree of conservation of each residue in all proteins.

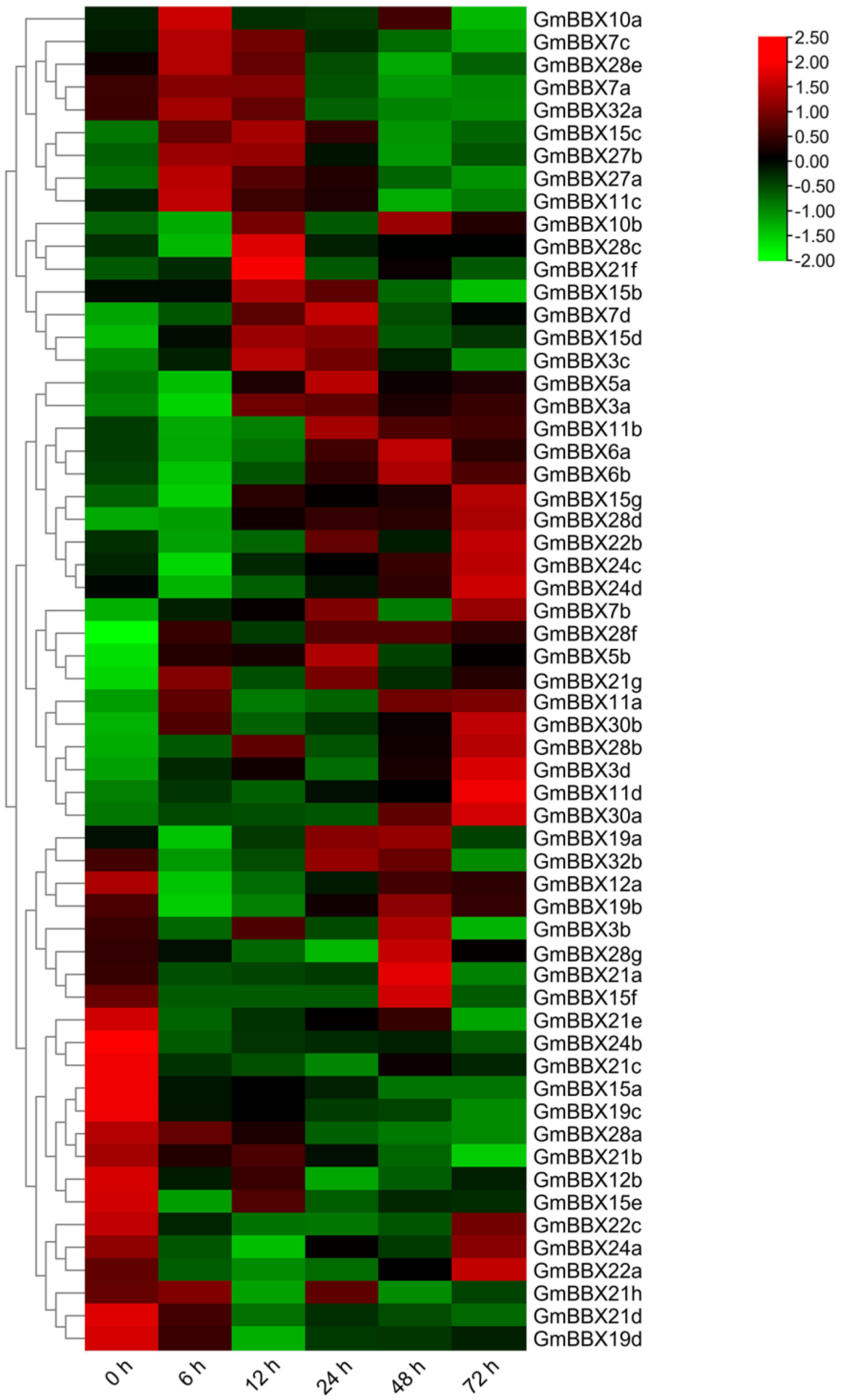

Fig. S4 The heatmap of the 59 *GmBBX* genes under salt stress using the online tool TBtools. Soybean seedlings were exposed to the salt stress of 200 mM NaCl for 0, 6, 12, 24, 48 and 72 h. The heatmap was generated with the FPKM values of the 59 salt-stress-responsive *GmBBX*s using the online tool, TBtools. The color scale beside the heat map indicates gene expression levels, low transcript abundance indicated by green color and high transcript abundance indicated by red color.

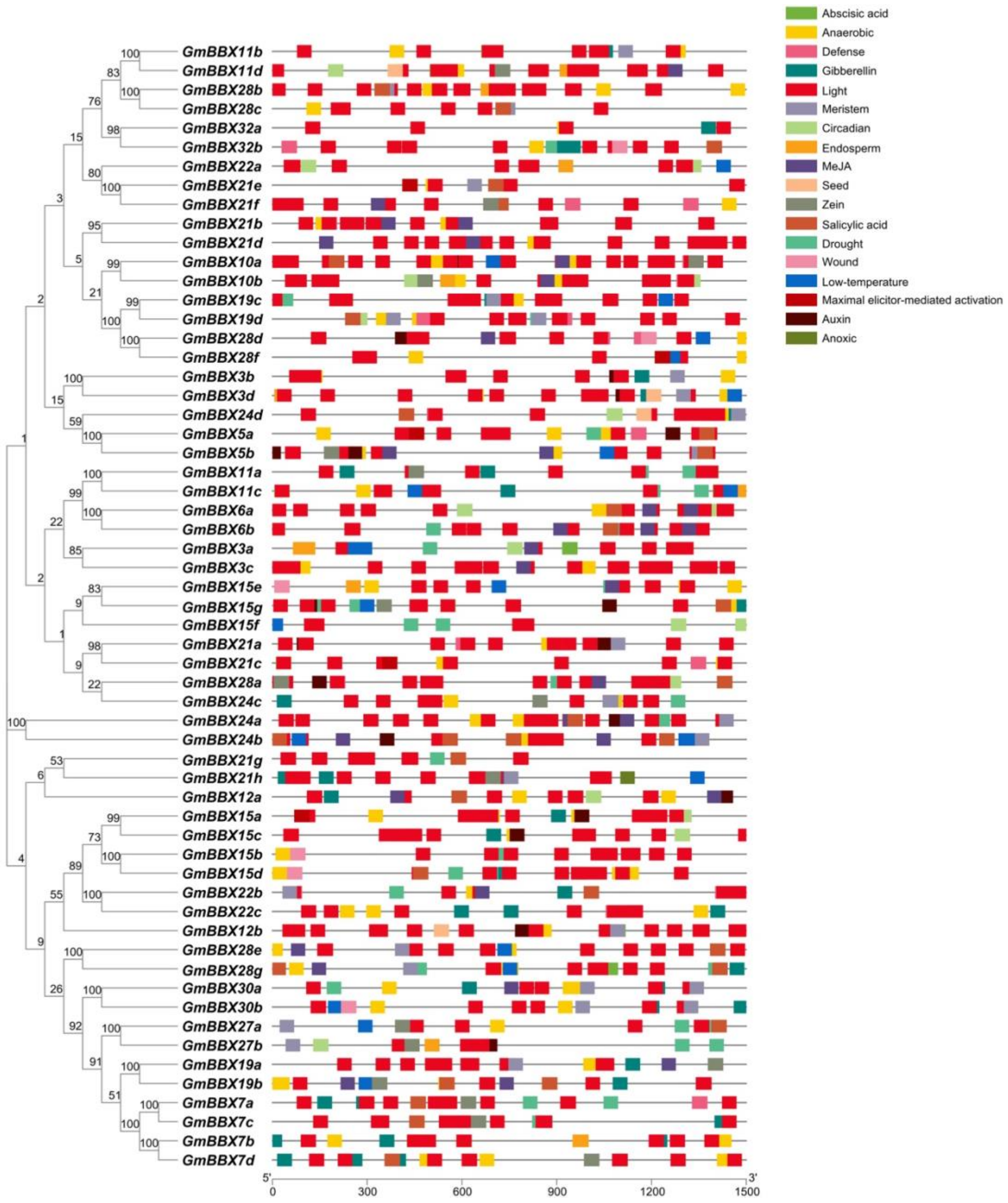

Fig. S5 The *cis*-acting elements in the promoter regions of the 59 *GmBBX* genes. 1,500 bp interval upstream of the translation initiation site of each *GmBBX* gene was considered as promoter region. The phylogenetic tree was generated using the promoter sequences of *GmBBX* genes (the left panel). Fifty-nine promoter sequences were applied for the prediction of *cis*-acting elements using the online program, PlantCARE. The colored boxes in the middle panel represent different *cis*-acting elements, and the sequence length of each promoter is represented by grey bar at the bottom. The symbols in the right panel are corresponding to the colored boxes.
